# Baseline predictors of visual response and treatment burden after ranibizumab therapy in macular edema secondary to retinal vein occlusion

**DOI:** 10.3389/fmed.2026.1856268

**Published:** 2026-06-10

**Authors:** Xudong Li, Lan Yang, He Long, Xin Li

**Affiliations:** 1 Department of Ophthalmology, Wuhan Aier Eye Hanyang Hospital, Wuhan, China; 2 Aier Eye Hospital Group Co., Ltd., Changsha, China

**Keywords:** anti-VEGF, inflammatory biomarkers, macular edema, OCT, retinal vein occlusion, treatment burden, visual outcome

## Abstract

**Background:**

Visual response to anti-vascular endothelial growth factor (anti-VEGF) therapy in macular edema secondary to retinal vein occlusion (RVO-ME) varies among patients, and baseline predictors remain incompletely defined. This study investigated baseline predictors of visual response and treatment burden after anti-VEGF therapy in eyes with RVO-ME.

**Methods:**

This retrospective observational study included 80 eyes from 80 patients with RVO-ME treated at a single center. Baseline clinical characteristics, optical coherence tomography (OCT) biomarkers, and hematologic inflammatory indices were collected. Decimal visual acuity was converted to approximate Early Treatment Diabetic Retinopathy Study (ETDRS) letter scores. Poor functional response was defined as a gain of ≤10 ETDRS letters at 1 month after the third anti-VEGF injection. A composite endpoint was used for sensitivity analysis. Treatment burden was assessed by the number of injections within 6 months.

**Results:**

Mean baseline and final ETDRS letter scores were 37.46 and 58.93, respectively, with a mean gain of 21.47 letters. Poor functional response occurred in 21 eyes (26.3%). After excluding sparse HRVO cases from regression modeling, CRVO was associated with poor functional response compared with BRVO (OR 6.81, 95% CI 1.17–39.74, *p* = 0.033), as were higher baseline ETDRS letter score per 10-letter increase (OR 1.85, 95% CI 1.20–2.84, *p* = 0.005) and male sex (OR 5.46, 95% CI 1.34–22.35, *p* = 0.018). Poor response was less frequent among patients with hypertension (OR 0.13, 95% CI 0.03–0.59, *p* = 0.008). Firth penalized logistic regression yielded similar results. In the continuous outcome model, CRVO was associated with lower final ETDRS letter score (β = −13.78 letters, 95% CI −19.62 to −7.93, *p* < 0.001). Conventional qualitative OCT biomarkers were not significantly associated with the primary gain-based endpoint, although CST showed stronger relevance in the composite endpoint analysis. In adjusted Poisson regression, CRVO showed a non-significant trend toward higher injection count.

**Conclusion:**

In this small retrospective RVO-ME cohort treated with ranibizumab, CRVO was consistently associated with poorer short-term visual outcome. The association between baseline visual acuity and gain-based response appeared to be influenced by a ceiling effect. Conventional qualitative OCT biomarkers and hematologic inflammatory indices showed limited but potentially complementary value, and treatment-burden findings should be interpreted as exploratory.

## Introduction

Retinal vein occlusion (RVO) is one of the most common retinal vascular disorders and a major cause of visual impairment worldwide (1). Macular edema is the leading cause of vision loss in patients with RVO, and intravitreal anti-vascular endothelial growth factor (anti-VEGF) therapy has become the standard first-line treatment for RVO-related macular edema (RVO-ME) (2–4). However, treatment response varies substantially across individuals. While some eyes achieve marked visual improvement after anti-VEGF therapy, others show limited functional recovery despite repeated injections (5, 6). Identifying baseline predictors of treatment response is therefore clinically important for prognosis estimation, individualized counseling, and treatment planning.

Previous studies have suggested that both local retinal structural changes and systemic factors may influence visual outcomes in RVO-ME (7). Optical coherence tomography (OCT) provides detailed information on retinal morphology and is widely used to evaluate disease severity and monitor treatment response. Several OCT-derived biomarkers, including central macular thickness (CST), disorganization of the inner retinal layers (DIRT), ellipsoid zone/external limiting membrane (EZ/ELM) disruption, intraretinal cysts (IRC), and subretinal fluid (SRF), have been investigated as potential predictors of prognosis in retinal vascular macular edema (7, 8). In RVO-related disease, baseline OCT biomarkers have been investigated as potential predictors of treatment outcome, and some studies have reported associations with visual or anatomical response. However, the reported findings have not been entirely consistent across cohorts, treatment regimens, and outcome definitions, and the prognostic value of conventional structural OCT parameters in routine clinical practice remains uncertain (3, 8, 9).

In addition to imaging findings, systemic inflammatory status may also contribute to the pathophysiology and treatment response of RVO. Increasing evidence suggests that inflammation, endothelial dysfunction, and microvascular dysregulation are involved in the development and progression of retinal vascular disease (10). Hematologic inflammatory indices derived from routine blood tests, such as neutrophil-to-lymphocyte ratio (NLR), platelet-to-lymphocyte ratio (PLR), systemic immune-inflammation index (SII), and monocyte-to-lymphocyte ratio (MLR), have attracted attention as inexpensive and readily available biomarkers. These indices have been investigated in several retinal vascular and macular edema conditions, but their role in predicting anti-VEGF response in RVO-ME has not been clearly established (11).

Another clinically relevant but less frequently addressed issue is treatment burden. In real-world practice, patients with RVO-ME often require repeated anti-VEGF injections during follow-up, yet the number of injections varies considerably between individuals (5). Baseline factors associated not only with visual response but also with subsequent injection burden may have practical implications for prognosis estimation, patient counseling, and follow-up planning. Evaluating treatment burden alongside functional outcome may therefore provide a more comprehensive understanding of disease behavior and therapeutic demand.

Our group has previously investigated the relative value of OCT-derived structural biomarkers and systemic inflammatory indicators in diabetic macular edema, and found that local retinal structural features appeared to show stronger short-term predictive value than peripheral inflammatory indices within a unified analytical framework (12). Whether a similar pattern exists in RVO-ME remains unclear. Given the differences in pathogenesis between diabetic macular edema and RVO-related macular edema, the relative contribution of local imaging biomarkers and systemic inflammatory factors may not be identical across disease entities.

Therefore, the present study aimed to investigate baseline predictors of functional outcome after anti-VEGF therapy in eyes with macular edema secondary to RVO, with particular attention to clinical characteristics, OCT structural biomarkers, and hematologic inflammatory indices. The primary endpoint was poor functional response, defined as a gain of ≤10 approximate ETDRS letters after treatment. A composite functional endpoint was additionally used in sensitivity analyses. Furthermore, we explored baseline factors associated with treatment burden, assessed by the number of anti-VEGF injections within 6 months.

## Methods

### Study design, setting, and ethical approval

This retrospective observational study was conducted at Wuhan Aier Eye Hanyang Hospital, Wuhan, China. Medical records of patients with RVO-ME who received intravitreal ranibizumab therapy at this center between June 2023 and June 2025 were retrospectively reviewed.

The study was conducted in accordance with the tenets of the Declaration of Helsinki and was approved by the Ethics Committee of Wuhan Aier Eye Hanyang Hospital (Approval No. IEC-AD-HYEYE2024008), which approved the retrospective review of medical records and waived the requirement for written informed consent. Only de-identified data were used for analysis.

### Study participants

Patients diagnosed with RVO-ME and treated with intravitreal ranibizumab therapy during the study period were screened for eligibility. All included patients had unilateral disease, and therefore one affected eye from each patient was included in the analysis. Only eyes with complete clinical, imaging, laboratory, treatment, and follow-up data for the variables of interest were included in the final analytical dataset. During the study period, 218 medical records of patients diagnosed with RVO-ME and treated with intravitreal therapy were screened. Eligibility was assessed according to predefined inclusion and exclusion criteria. A total of 138 records were excluded because of incomplete baseline or follow-up data (*n* = 32), poor-quality OCT images (*n* = 2), non-ranibizumab anti-VEGF treatment (*n* = 69), evidence of retinal vasculitis or other ocular inflammatory vascular disorders (*n* = 21), overt systemic infectious or inflammatory conditions at baseline (*n* = 5), or other reasons (*n* = 9). Finally, 80 eyes from 80 patients were included in the analysis. The patient selection process is summarized in Supplementary Figure S1.

Eyes were excluded if they had incomplete baseline or follow-up data, poor-quality OCT images precluding reliable biomarker grading, non-ranibizumab anti-VEGF treatment during the study period, or evidence of retinal vasculitis or other ocular inflammatory vascular disorders based on clinical examination and angiographic evaluation. Patients with overt systemic infectious or inflammatory conditions documented at baseline were also not included.

Patients were classified according to RVO subtype as BRVO, CRVO, or HRVO. Eyes recorded as superotemporal or inferotemporal vein occlusion were categorized as BRVO, eyes recorded as central vein occlusion were categorized as CRVO, and eyes recorded as superior or inferior hemispheric vein occlusion were categorized as HRVO. A total of 80 eyes from 80 patients were eligible for the final analysis.

### Treatment regimen

All included eyes received intravitreal ranibizumab injections. The treatment protocol consisted of an initial three-injection loading phase followed by a pro re nata (PRN) regimen. Retreatment decisions during follow-up were based on routine clinical assessment, including persistent or recurrent macular edema on OCT, insufficient visual improvement, or visual decline. Treatment burden was evaluated by the total number of ranibizumab injections administered within 6 months.

### Data collection

Baseline demographic and clinical variables included age, sex, RVO subtype, disease duration, hypertension, diabetes mellitus, and the number of ranibizumab injections within 6 months. Disease duration was determined according to the recorded duration from patient-reported symptom onset to the first treatment visit. Because symptom onset in RVO may be uncertain, this variable was interpreted cautiously.

Best-corrected visual acuity (BCVA) was recorded at baseline, 1 month after the initial visit, and 1 month after the third ranibizumab injection. Baseline OCT variables included CST, DIRT, EZ/ELM disruption, IRC, and SRF.

Baseline hematologic variables were obtained from routine blood tests performed before initiation of intravitreal ranibizumab treatment. These variables included white blood cell count, neutrophil count, lymphocyte count, monocyte count, and platelet count. Derived inflammatory indices, including NLR, PLR, SII, and MLR, were calculated according to standard definitions and analyzed as exploratory systemic inflammatory biomarkers.

### Visual acuity conversion and outcome definitions

Visual acuity was originally documented as decimal visual acuity. For statistical standardization, decimal visual acuity was converted to logMAR, and approximate ETDRS letter scores were subsequently derived using a commonly used conversion method. These ETDRS letter scores should therefore be interpreted as approximate values rather than direct ETDRS chart measurements.

The primary functional endpoint was poor functional response, defined as a gain of ≤10 approximate ETDRS letters from baseline to 1 month after the third ranibizumab injection. Eyes with a gain of >10 approximate ETDRS letters were classified as good responders.

A composite endpoint was used as a sensitivity analysis to reduce the influence of the ceiling effect inherent in gain-based endpoints. Eyes were considered to have a favorable composite outcome if they achieved either a gain of ≥10 approximate ETDRS letters or a final decimal visual acuity of ≥0.3. Eyes that achieved neither criterion were classified as having a poor composite outcome.

To further address concerns regarding the ceiling effect, final approximate ETDRS letter score was also analyzed as a continuous outcome in additional multivariable linear regression models adjusted for baseline visual acuity and other clinically relevant covariates.

### OCT image acquisition and assessment

Baseline OCT images were obtained using the Zeiss CIRRUS HD-OCT 5000 system. CST was defined as the machine-measured central macular thickness recorded by the OCT device. Qualitative OCT biomarkers, including DIRT, EZ/ELM disruption, IRC, and SRF, were independently assessed by two retinal specialists who were masked to treatment response and injection burden. Disagreements were resolved by discussion and consensus.

Intergrader agreement for qualitative OCT biomarkers was evaluated using Cohen’s *κ*. The final consensus results were used in the main statistical analyses. The OCT acquisition and grading framework was consistent with the imaging assessment approach used in our previous retinal biomarker study (12).

### Treatment burden

Treatment burden was evaluated by the number of ranibizumab injections administered within 6 months. This variable was analyzed as both a continuous count outcome and a categorical exploratory outcome. For descriptive purposes, high treatment burden was defined as receiving ≥4 injections within 6 months, whereas low treatment burden was defined as receiving ≤3 injections. Because all eyes received an initial three-injection loading phase, the binary high-burden classification was interpreted cautiously and considered exploratory.

### Statistical analysis

Continuous variables were expressed as mean ± standard deviation or median with interquartile range, as appropriate. Categorical variables were presented as counts and percentages. Between-group comparisons were performed using the independent-samples *t* test or Mann–Whitney *U* test for continuous variables, and the chi-square test or Fisher’s exact test for categorical variables.

Univariate logistic regression analyses were first performed to explore baseline factors associated with poor functional response. Candidate variables included age, sex, disease duration, RVO subtype, hypertension, diabetes mellitus, baseline approximate ETDRS letter score, CST, DIRT, EZ/ELM disruption, IRC, SRF, NLR, PLR, SII, and MLR.

Because HRVO cases were few and generated unstable estimates due to sparse-data separation, HRVO eyes were excluded from regression modeling and retained only in descriptive analyses. Revised logistic regression models were therefore fitted in eyes with BRVO or CRVO. The main multivariable logistic model included clinically relevant covariates selected *a priori*: baseline approximate ETDRS letter score, RVO subtype, hypertension, sex, and NLR. Baseline ETDRS letter score was scaled per 10-letter increase to improve clinical interpretability. ORs and 95% CIs were reported.

Given the modest number of poor-response events and the risk of sparse-data bias, Firth penalized logistic regression was performed as a sensitivity analysis for the main binary endpoint. Model discrimination was assessed using the area under the receiver operating characteristic curve (AUC), calibration was evaluated using the Hosmer-Lemeshow goodness-of-fit test, and multicollinearity was assessed using variance inflation factors (VIFs). As an internal validation procedure, bootstrap resampling was performed for the revised multivariable logistic regression model to estimate optimism-corrected discrimination. In each bootstrap sample, the model was refitted, and the difference between bootstrap performance and test performance in the original dataset was used to estimate optimism. The optimism-corrected AUC was calculated by subtracting the average optimism from the apparent AUC.

To address the ceiling-effect concern associated with the gain-based primary endpoint, additional multivariable linear regression models were constructed using final approximate ETDRS letter score and ETDRS letter gain as continuous outcomes. These models were adjusted for baseline approximate ETDRS letter score, RVO subtype, hypertension, sex, CST, and NLR. CST was scaled per 50 μm for clinical interpretability.

Sensitivity analyses were performed using the composite functional endpoint. Because the number of poor composite outcomes was limited, results from multivariable models for this endpoint were interpreted cautiously.

For treatment burden, Spearman correlation analysis was first used to evaluate associations between injection number and continuous baseline variables. Differences in injection number across categorical baseline variables were assessed using the Mann–Whitney *U* test or Kruskal-Wallis test, as appropriate. In addition, injection count within 6 months was analyzed using count regression models. Poisson regression was used when no overdispersion was evident, and negative binomial regression was explored if overdispersion was present. Covariates in the adjusted count model included RVO subtype, baseline approximate ETDRS letter score, CST, PLR, and hypertension. IRRs and 95% CIs were reported. PLR was scaled per 50-unit increase.

All analyses were considered exploratory and hypothesis-generating. Two-sided *p* values <0.05 were considered statistically significant, whereas borderline findings were interpreted cautiously in the context of multiple exploratory comparisons and the limited sample size. Statistical analyses were performed using Python with the pandas, NumPy, SciPy, statsmodels, scikit-learn, and openpyxl packages.

## Results

### Baseline characteristics

Of 218 screened medical records, 80 eyes from 80 patients with RVO-ME met the eligibility criteria and were included in the final analysis. All included eyes had complete clinical, imaging, laboratory, treatment, and follow-up data for the variables of interest; therefore, no missing data were present in the final analytical dataset.

After conversion of decimal visual acuity to approximate ETDRS letter scores, the mean baseline ETDRS letter score was 37.46, and the mean ETDRS letter score at 1 month after the third ranibizumab injection was 58.93, corresponding to a mean gain of 21.47 letters. Based on the predefined primary endpoint, 21 eyes (26.3%) were classified as poor responders, whereas 59 eyes (73.7%) were classified as good responders. According to the composite endpoint, 10 eyes (12.5%) failed to achieve either a gain of ≥10 approximate ETDRS letters or a final decimal visual acuity of ≥0.3. In addition, 50 eyes (62.5%) received at least 4 ranibizumab injections within 6 months (Table 1).

**Table 1:** Baseline characteristics of the study population according to primary functional outcome.

Variable	Good response (*n* = 59)	Poor response (*n* = 21)	*p* value
Age, years	61.00 (54.00, 69.00)	61.00 (57.00, 71.00)	0.722
Disease duration, days	30.00 (10.00, 60.00)	30.00 (14.00, 60.00)	0.691
Baseline ETDRS letters	38.96 (21.93, 52.47)	47.45 (31.93, 61.98)	0.109
Central macular thickness, μm	552.50 (394.25, 664.25)	548.50 (411.00, 731.00)	0.956
NLR	2.30 (1.72, 2.87)	2.74 (2.15, 3.46)	0.077
PLR	134.34 (111.55, 172.24)	145.26 (116.19, 184.40)	0.592
SII	477.40 (362.04, 665.12)	591.94 (439.71, 756.73)	0.193
MLR	0.20 (0.16, 0.25)	0.22 (0.17, 0.28)	0.559
Number of injections within 6 months	4.00 (3.00, 4.00)	4.00 (4.00, 5.00)	0.093
Female sex, *n* (%)	35 (59.3)	8 (38.1)	0.155
Male sex, *n* (%)	24 (40.7)	13 (61.9)	0.155
RVO subtype, *n* (%)			0.033
BRVO	39 (66.1)	9 (42.9)	
CRVO	16 (27.1)	12 (57.1)	
HRVO	4 (6.8)	0 (0.0)	
Hypertension, *n* (%)	45 (76.3)	8 (38.1)	0.004
Diabetes mellitus, *n* (%)	13 (22.0)	2 (9.5)	0.331
DIRT present, *n* (%)	29 (49.2)	9 (42.9)	0.809
EZ/ELM disruption, *n* (%)	25 (42.4)	8 (38.1)	0.933
IRC present, *n* (%)	36 (61.0)	12 (57.1)	0.959
SRF present, *n* (%)	25 (42.4)	5 (23.8)	0.213

Data are presented as median (interquartile range) or *n* (%), as appropriate. Poor functional response was defined as a gain of ≤10 ETDRS letters after anti-VEGF therapy.

BRVO, branch retinal vein occlusion; CRVO, central retinal vein occlusion; HRVO, hemi-retinal vein occlusion; DIRT, disorganization of the inner retinal layers; EZ/ELM, ellipsoid zone/external limiting membrane; IRC, intraretinal cysts; SRF, subretinal fluid; NLR, neutrophil-to-lymphocyte ratio; PLR, platelet-to-lymphocyte ratio; SII, systemic immune-inflammation index; MLR, monocyte-to-lymphocyte ratio.

### Reproducibility of OCT biomarker grading

Qualitative OCT biomarkers were independently assessed by two masked retinal specialists, and the final consensus results were used in the main analyses. Intergrader agreement was excellent for all qualitative OCT biomarkers, with Cohen’s κ values of 0.899 for DIRT, 0.949 for EZ/ELM disruption, 0.974 for IRC, and 0.973 for SRF.

### Baseline comparisons between poor and good responders

There were no significant differences between poor and good responders in age, disease duration, baseline CST, or most OCT structural parameters. Median age did not differ significantly between the two groups (*p* = 0.722), nor did disease duration (*p* = 0.691). Baseline CST was also not associated with the primary endpoint (*p* = 0.956). Likewise, DIRT (*p* = 0.809), EZ/ELM disruption (*p* = 0.933), IRC (*p* = 0.959), and SRF (*p* = 0.213) were not significantly different between groups. Disease duration, whether analyzed as ≤30 versus >30 days (*p* = 0.394) or categorized into four groups (≤7, 8–14, 15–30, and >30 days), was not associated with poor response (*p* = 0.515) (Table 1).

In contrast, RVO subtype and hypertension status differed significantly between groups. Poor response was more frequent in CRVO than in BRVO or HRVO (*p* = 0.033). Poor response was less frequent in patients with hypertension than in those without hypertension (*p* = 0.004). Sex showed a non-significant trend, with poor response occurring more often in male patients (*p* = 0.155). Diabetes mellitus was not associated with treatment response (*p* = 0.331) (Table 1).

### Hematologic markers and the primary endpoint

Among hematologic markers, NLR showed a borderline association with poor functional response (*p* = 0.077). In contrast, PLR (*p* = 0.592), SII (*p* = 0.193), and MLR (*p* = 0.559) were not significantly associated with the primary endpoint. The number of injections within 6 months also showed a borderline difference between groups (*p* = 0.093) (Table 1).

### Univariate and revised multivariable logistic regression for poor functional response

In univariate logistic regression analyses, hypertension was significantly associated with the primary endpoint (*p* = 0.002), and CRVO subtype was associated with a higher likelihood of poor response compared with BRVO (*p* = 0.027). Baseline ETDRS letter score (*p* = 0.084), male sex (*p* = 0.098), SRF (*p* = 0.137), and NLR (*p* = 0.164) showed suggestive but non-significant associations. No significant associations were observed for age, disease duration, diabetes mellitus, CST, DIRT, EZ/ELM disruption, IRC, PLR, SII, or MLR (Table 2).

**Table 2:** Univariate logistic regression analysis for poor functional response after anti-VEGF therapy.

Variable	OR	95% CI	*p* value
Hypertension	0.19	0.07–0.56	0.002
CRVO vs. BRVO	3.25	1.15–9.21	0.027
Baseline ETDRS (per letter)	1.02	1.00–1.05	0.084
Male sex	2.37	0.85–6.59	0.098
SRF	0.43	0.14–1.31	0.137
NLR	1.37	0.88–2.13	0.164
Diabetes mellitus	0.37	0.08–1.81	0.221
SII	1.001	0.999–1.002	0.351
Disease duration	1.006	0.992–1.020	0.405
PLR	1.003	0.996–1.010	0.435
CST	1.001	0.999–1.002	0.512
DIRT	0.78	0.28–2.12	0.620
EZ/ELM disruption	0.84	0.30–2.32	0.733
IRC	0.85	0.31–2.34	0.756
MLR	1.77	0.01–295.91	0.826
Age	1.00	0.96–1.04	0.971

Poor functional response was defined as a gain of ≤10 approximate ETDRS letters after ranibizumab therapy. BRVO was used as the reference category for RVO subtype. Analyses were based on 80 eyes unless otherwise indicated. For RVO subtype, BRVO was used as the reference category, and HRVO was not estimated because sparse events generated unstable estimates.

OR, odds ratio; CI, confidence interval; ETDRS, Early Treatment Diabetic Retinopathy Study; SRF, subretinal fluid; NLR, neutrophil-to-lymphocyte ratio; SII, systemic immune-inflammation index; PLR, platelet-to-lymphocyte ratio; CST, central macular thickness; DIRT, disorganization of the inner retinal layers; EZ/ELM, ellipsoid zone/external limiting membrane; IRC, intraretinal cysts; MLR, monocyte-to-lymphocyte ratio.

**Figure 1:**
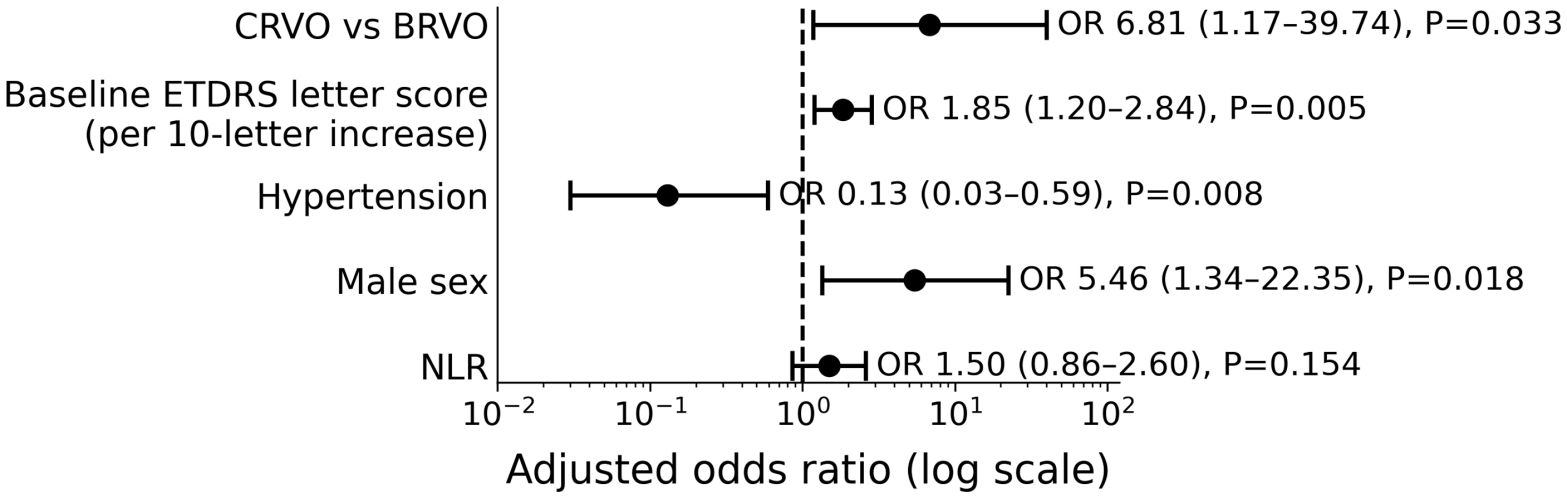
Revised multivariable predictors of poor functional response. HRVO eyes were excluded from regression modeling because of sparse events and separation concerns; *n* = 76. Model discrimination: apparent AUC = 0.866; optimism-corrected AUC = 0.828. Additional diagnostics: Hosmer-Lemeshow *p* = 0.439; VIF 1.04–1.42.

Because HRVO cases were few and generated unstable estimates due to sparse-data separation in logistic regression, HRVO eyes were excluded from revised regression modeling and retained only in descriptive analyses. Among BRVO and CRVO eyes, the revised multivariable logistic regression model included 76 eyes, including 21 poor-response events. CRVO remained associated with poor functional response compared with BRVO (OR 6.81, 95% CI 1.17–39.74, *p* = 0.033). Higher baseline ETDRS letter score, scaled per 10-letter increase, was also associated with poor response (OR 1.85, 95% CI 1.20–2.84, *p* = 0.005). Male sex was associated with a higher likelihood of poor response (OR 5.46, 95% CI 1.34–22.35, *p* = 0.018). In this cohort, poor functional response was less frequent among patients with hypertension than among those without hypertension (OR 0.13, 95% CI 0.03–0.59, *p* = 0.008). NLR was not significantly associated with poor response in the revised model (OR 1.50, 95% CI 0.86–2.60, *p* = 0.154) (Table 3; Figure 1).

**Table 3:** Revised multivariable logistic regression analysis for poor functional response after anti-VEGF therapy.

Variable	OR	95% CI	*p* value
CRVO vs. BRVO	6.81	1.17–39.74	0.033
Baseline ETDRS (per 10 letters)	1.85	1.20–2.84	0.005
Hypertension	0.13	0.03–0.59	0.008
Male sex	5.46	1.34–22.35	0.018
NLR	1.50	0.86–2.60	0.154

HRVO eyes were excluded from regression modeling because of sparse events and separation concerns; *n* = 76. Model diagnostics: apparent AUC = 0.866; optimism-corrected AUC = 0.828; Hosmer-Lemeshow *p* = 0.439; VIF 1.04–1.42.

OR, odds ratio; CI, confidence interval; ETDRS, Early Treatment Diabetic Retinopathy Study; NLR, neutrophil-to-lymphocyte ratio.

The revised logistic model showed acceptable discrimination, with an apparent AUC of 0.866. Bootstrap internal validation using 1,000 resamples showed a mean optimism of 0.038 and an optimism-corrected AUC of 0.828, with an approximate bootstrap interval of 0.748–0.913. The Hosmer-Lemeshow goodness-of-fit test did not indicate lack of fit (*p* = 0.439). VIFs ranged from approximately 1.04–1.42, indicating no evidence of problematic multicollinearity.

### Firth penalized logistic regression sensitivity analysis

Given the modest number of poor-response events, Firth penalized logistic regression was performed as a sensitivity analysis. The results were generally consistent with the revised conventional logistic model. CRVO remained associated with poor functional response compared with BRVO (OR 5.42, 95% CI 1.08–27.30, *p* = 0.040). Baseline ETDRS letter score per 10-letter increase was also associated with poor response (OR 1.70, 95% CI 1.15–2.52, *p* = 0.008), as was male sex (OR 4.38, 95% CI 1.21–15.93, *p* = 0.025). Poor response was less frequent among patients with hypertension (OR 0.17, 95% CI 0.04–0.66, *p* = 0.010). NLR remained non-significant (OR 1.43, 95% CI 0.87–2.36, *p* = 0.162) (Supplementary Table S3).

### Continuous visual outcome analysis

To address the ceiling-effect concern associated with the gain-based primary endpoint, final ETDRS letter score was additionally analyzed as a continuous outcome in a multivariable linear regression model adjusted for baseline ETDRS letter score and other clinically relevant covariates. This model included 76 BRVO and CRVO eyes and showed good explanatory performance (*R*^2^ = 0.744; adjusted *R*^2^ = 0.722).

In this model, baseline ETDRS letter score was positively associated with final ETDRS letter score (β = 0.49 per letter, 95% CI 0.37–0.62, *p* < 0.001), whereas CRVO was associated with a lower final ETDRS letter score compared with BRVO (β = −13.78 letters, 95% CI −19.62 to −7.93, *p* < 0.001). Hypertension (β = 3.62, *p* = 0.173) and male sex (β = −2.08, *p* = 0.377) were not significantly associated with final ETDRS letter score in this continuous outcome model. CST showed a non-significant trend toward lower final ETDRS letter score when scaled per 50 μm increase (β = −0.40, *p* = 0.088), and NLR also showed a borderline association (β = −2.07, *p* = 0.056) (Supplementary Table S4).

An additional model using ETDRS letter gain as a continuous outcome showed that higher baseline ETDRS letter score was associated with smaller letter gain, supporting the presence of a ceiling effect in gain-based outcome definitions.

### Sensitivity analysis using the composite endpoint

When the composite endpoint was applied, CST showed a stronger univariate association with poor outcome (*p* = 0.003). CRVO subtype (*p* = 0.004), hypertension (*p* = 0.017), PLR (*p* = 0.028), and baseline ETDRS letter score (*p* = 0.038) were also significantly associated with the composite outcome in univariate analysis. NLR (*p* = 0.057), SII (*p* = 0.059), and EZ/ELM disruption (*p* = 0.061) showed borderline significance. However, because only 10 eyes met the composite poor-outcome definition, multivariable modeling for this endpoint was considered unstable and was interpreted cautiously (Supplementary Table S1).

### Analysis of treatment burden

Exploratory analyses were performed to evaluate treatment burden during the first 6 months. In Spearman correlation analyses, PLR was positively correlated with the number of injections (rho = 0.240, *p* = 0.032), whereas baseline ETDRS letter score was negatively correlated with injection number (rho = −0.229, *p* = 0.041). CST showed a borderline positive correlation with injection number (rho = 0.215, *p* = 0.055), and SII also showed a weak trend (rho = 0.193, *p* = 0.086). No significant correlations were found for age, disease duration, NLR, or MLR (Supplementary Table S2A).

**Figure 2:**
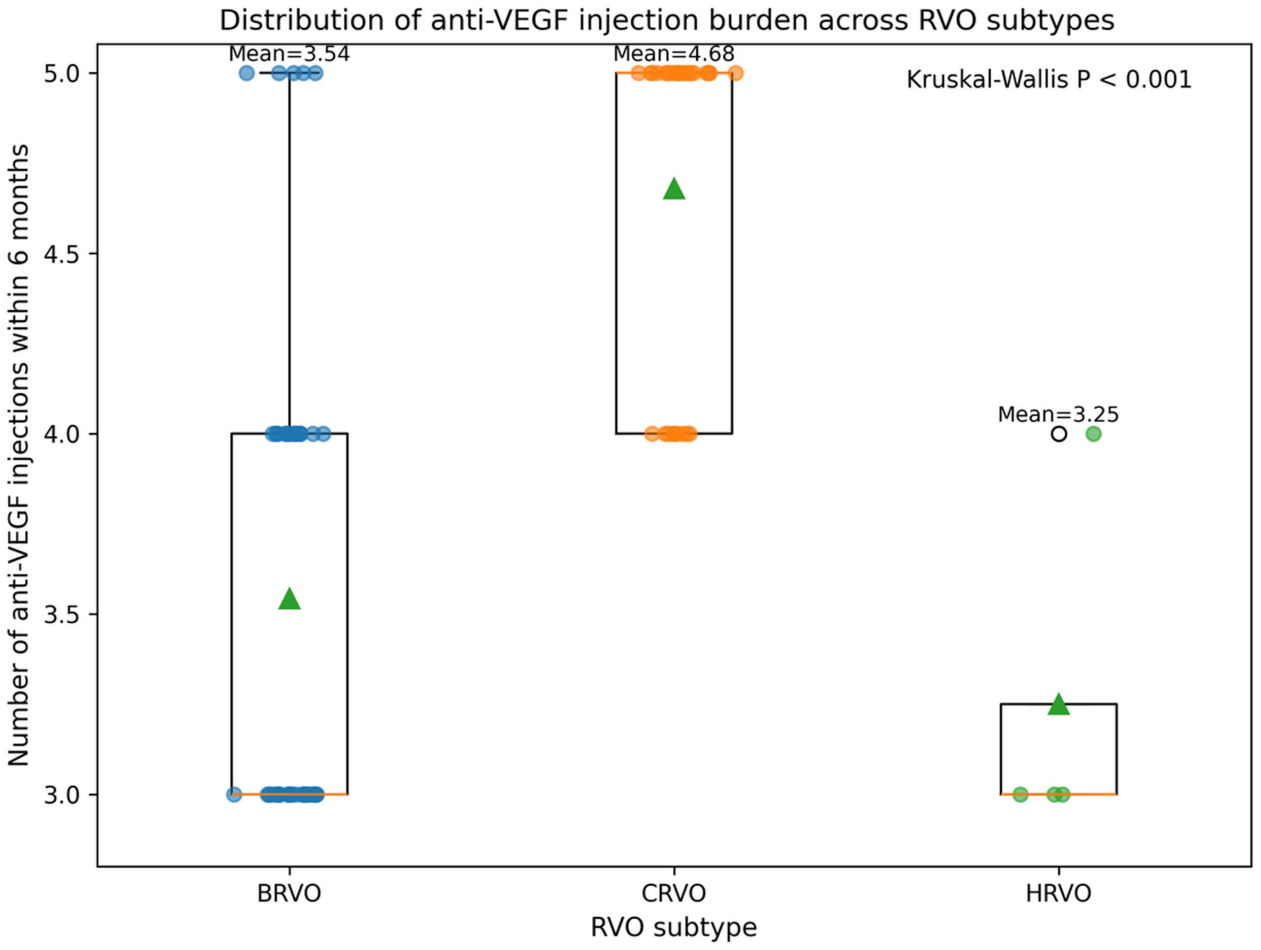
Distribution of anti-VEGF injection burden across RVO subtypes.

The number of injections differed significantly across RVO subtypes (*p* < 0.001). Eyes with CRVO received more injections on average than eyes with BRVO (mean 4.68 vs. 3.54), whereas HRVO eyes received a mean of 3.25 injections. Injection number also differed by hypertension status (*p* = 0.002), with patients without hypertension receiving more injections than those with hypertension. No significant differences in injection number were observed for diabetes mellitus, DIRT, EZ/ELM disruption, IRC, SRF, or disease duration categorized at 30 days (Figure 2; Supplementary Table S2B).

Because injection number is a count outcome, an adjusted count regression model was additionally performed among BRVO and CRVO eyes. There was no evidence of overdispersion in injection count; therefore, Poisson regression was used for the adjusted analysis. In this model, CRVO showed a non-significant trend toward a higher injection count compared with BRVO (IRR 1.28, 95% CI 0.96–1.71, *p* = 0.092). Baseline ETDRS letter score per 10-letter increase (IRR 1.00, *p* = 0.918), CST per 50 μm increase (IRR 1.00, *p* = 0.991), PLR per 50-unit increase (IRR 1.00, *p* = 0.938), and hypertension (IRR 0.94, *p* = 0.626) were not independently associated with injection count (Supplementary Table S5). Therefore, treatment-burden findings were interpreted as exploratory.

## Discussion

In this retrospective study of eyes with RVO-ME treated with ranibizumab, several baseline clinical factors were associated with short-term gain-based visual response, whereas conventional qualitative OCT biomarkers showed limited predictive value for the primary endpoint. The main findings were as follows: first, CRVO was associated with poor functional response compared with BRVO in the revised logistic model excluding sparse HRVO cases, and this association remained generally consistent in Firth penalized logistic regression; second, CRVO was also associated with lower final ETDRS letter score in the continuous visual outcome model; third, the association between higher baseline ETDRS letter score and poor gain-based response appeared to be strongly influenced by the ceiling effect inherent in the primary endpoint; fourth, hypertension and male sex were associated with the binary gain-based endpoint but not with final ETDRS letter score, suggesting that these findings should be interpreted cautiously; and fifth, treatment-burden analyses were exploratory, with CRVO showing a non-significant trend toward higher injection count after adjustment.

One of the most clinically relevant findings of this study was the association between CRVO and poorer visual outcome. Compared with BRVO, CRVO was associated with a substantially higher likelihood of failing to achieve a gain of more than 10 approximate ETDRS letters. This finding is biologically plausible and consistent with the generally more severe disease profile of CRVO. CRVO often involves a larger retinal territory, more extensive venous outflow impairment, greater ischemic burden, and a higher VEGF-driven exudative load than BRVO, which may help explain its less favorable functional response and greater therapeutic demand in clinical practice (5, 13). Importantly, CRVO was also associated with lower final ETDRS letter score in the continuous outcome model, suggesting that the subtype-related association was not solely driven by the gain-based endpoint definition. Accordingly, RVO subtype appears to be a clinically relevant baseline factor for prognosis estimation, although the present findings require validation in larger prospective cohorts.

Higher baseline ETDRS letter score was associated with poor functional response in the binary gain-based model. However, this association should be interpreted primarily as an endpoint-related ceiling effect rather than as evidence of reduced biological responsiveness to ranibizumab. Eyes with better baseline vision have less room for measurable letter gain and are therefore more likely to be classified as poor responders when response is defined solely by a gain threshold; this issue is also relevant to the interpretation of outcomes in RVO eyes with relatively good baseline visual acuity (14). Therefore, baseline visual acuity should be interpreted as an important contextual and adjustment variable rather than as a straightforward marker of poor treatment responsiveness.

Male sex was associated with poor response in the revised logistic model and in the Firth sensitivity analysis, but it was not significantly associated with final ETDRS letter score in the continuous outcome model. This finding should therefore be interpreted cautiously. Sex may capture unmeasured differences in vascular risk profile, presentation timing, ischemic status, treatment adherence, or other systemic background factors. Given the limited sample size and exploratory nature of the analysis, this association should be regarded as hypothesis-generating and requires further validation.

An unexpected finding was that poor functional response was observed less frequently among patients with hypertension than among those without hypertension in the binary gain-based model. This direction does not fully align with the usual assumption that systemic vascular comorbidity would necessarily be associated with worse ocular prognosis. We rechecked the raw data and found no evidence of coding error. However, the association was not significant in the continuous final ETDRS model and should not be interpreted as evidence that hypertension is protective. Several explanations may account for this observation, including residual confounding, differences in systemic treatment patterns, medical follow-up intensity, RVO subtype distribution, or unmeasured ischemic and inflammatory factors. The non-hypertensive subgroup may also have included a higher proportion of patients in whom inflammatory, hematologic, or other non-hemodynamic mechanisms contributed to RVO; previous studies have suggested links between RVO and peripheral inflammatory biomarker profiles (10, 15). Overall, the hypertension finding should be considered exploratory and requires independent validation.

In contrast to the clinical variables, conventional qualitative OCT structural biomarkers showed limited predictive value for the primary gain-based endpoint in the present cohort. Neither DIRT, EZ/ELM disruption, IRC, SRF, nor baseline CST was significantly associated with poor response in the primary grouped comparisons or in univariate analyses for the gain-based endpoint. Previous studies have suggested that some baseline OCT biomarkers may be associated with visual or anatomical outcomes after anti-VEGF therapy in RVO-ME, although the specific biomarkers identified and their reported prognostic significance have varied across studies (3, 8, 16). The present null findings should not be interpreted as evidence that retinal morphology is unimportant. Rather, they may reflect limited statistical power, binary rather than graded biomarker assessment, short-term follow-up, lack of ischemia/perfusion data, and the use of a gain-based endpoint vulnerable to ceiling effects.

The OCT findings were more nuanced when alternative endpoints were considered. Under the composite outcome definition, CST showed a stronger association with poor outcome, and EZ/ELM disruption approached borderline significance. Similarly, in the continuous final ETDRS model, CST showed a non-significant trend toward lower final ETDRS score. These results suggest that OCT structural features may be more relevant for endpoints incorporating final visual status or longer-term anatomical persistence than for a single short-term letter-gain threshold. Therefore, in this small short-term cohort, conventional qualitative OCT biomarkers did not independently predict the primary gain-based endpoint, but structural OCT assessment may still provide complementary prognostic information depending on outcome definition and follow-up duration.

This finding should also be interpreted in the context of our previous work in diabetic macular edema, in which OCT-derived structural biomarkers showed stronger short-term predictive value for anti-VEGF response than several systemic inflammatory indicators when both categories of variables were evaluated within the same analytical framework (12, 17). In that cohort, DIRT and EZ/ELM disruption emerged as important predictors, whereas peripheral inflammatory indices showed limited independent predictive value. In the present RVO-ME cohort, however, the pattern was not identical. This discrepancy may reflect differences in disease pathophysiology. DME is often characterized by chronic neurovascular and metabolic injury, whereas RVO-ME may have a more acute vascular occlusive component, with ischemic burden and venous outflow impairment playing major roles. These differences may partly explain why qualitative OCT biomarkers were less discriminative for short-term letter gain in the present RVO-ME cohort.

The hematologic inflammatory markers in this study showed limited but potentially complementary value. NLR exhibited a borderline association with poor functional response in the primary analysis but did not remain independently significant in the revised logistic or Firth models. PLR and SII were not significantly associated with the primary endpoint, although PLR was correlated with injection number in the exploratory correlation analysis. Previous studies have reported altered peripheral inflammatory biomarker profiles in RVO, although their independent prognostic value for treatment response has not been consistent across studies (10, 15, 18). Taken together, these findings suggest that systemic inflammatory status may contribute to disease activity or treatment demand, but its predictive strength is likely modest and disease-dependent. The observed PLR–injection association should not be considered clinically actionable without validated cutoffs and prospective replication.

Beyond visual outcome, the present study explored treatment burden during the first 6 months under a 3 + PRN regimen. In descriptive analyses, CRVO eyes received more injections than BRVO eyes, and injection number differed across RVO subtypes. However, after adjustment in the Poisson count model, CRVO showed only a non-significant trend toward a higher injection count, and PLR was not independently associated with injection number. These findings indicate that early injection requirements may vary by disease subtype, although the adjusted count model did not confirm an independent association. Injection number within 6 months may reflect not only disease activity but also protocol structure, physician decision-making, patient adherence, and follow-up patterns. Therefore, the treatment-burden results should be interpreted as hypothesis-generating rather than confirmatory.

This study has several limitations. First, it was a single-center retrospective study with a relatively small sample size, and all multivariable models should be considered exploratory. Second, although HRVO cases were retained in descriptive analyses, they were excluded from regression modeling because of sparse events and separation concerns, which limits inference for this subgroup. Third, the primary endpoint was based on letter gain and was vulnerable to a ceiling effect; although we added continuous visual outcome models and a composite endpoint to address this issue, approximate ETDRS scores were derived from decimal visual acuity rather than measured directly using ETDRS charts. Fourth, retinal ischemia and perfusion metrics, including fluorescein angiography-based non-perfusion area or OCT angiography parameters, were not included. Ischemic burden is an important unmeasured confounder in RVO-ME and may influence both visual prognosis and treatment demand. Fifth, OCT biomarkers were assessed as conventional qualitative variables, and more detailed graded or quantitative imaging features were not evaluated. Sixth, injection number within 6 months reflects early treatment burden under a 3 + PRN regimen and may be affected by clinical decision-making and follow-up adherence. Finally, although only complete cases were included in the final analysis, the retrospective design may have introduced selection bias.

Despite these limitations, the study has several strengths. It evaluated clinical characteristics, OCT biomarkers, hematologic inflammatory indices, and treatment burden within a homogeneous ranibizumab-treated real-world cohort. The inclusion of a continuous final visual outcome model, Firth penalized logistic regression, count regression for injection burden, and OCT intergrader agreement assessment strengthened the robustness and transparency of the revised analysis. In addition, the use of both gain-based and final-status-based endpoints provided a more balanced interpretation of short-term functional response.

In conclusion, in this small retrospective RVO-ME cohort treated with ranibizumab, CRVO was consistently associated with poorer short-term visual outcome compared with BRVO, including in both revised binary and continuous outcome analyses. The association between higher baseline ETDRS letter score and poor gain-based response appeared to be strongly influenced by a ceiling effect. Conventional qualitative OCT biomarkers did not independently predict the primary gain-based endpoint, although CST and outer retinal integrity may be more relevant when endpoints incorporate final visual status. Associations involving hypertension, sex, inflammatory indices, and injection burden should be considered exploratory. Larger prospective studies incorporating standardized ETDRS measurement, perfusion/ischemia metrics, longer follow-up, and externally validated models are needed to confirm these findings and improve individualized prediction of visual outcome and treatment demand.

## Data Availability

The de-identified individual-level data supporting the findings of this study and the analysis code used for the revised analyses are available from the corresponding author upon reasonable request. Access will be subject to approval by the study institution and applicable ethical requirements. The data are not publicly deposited because they were derived from retrospective clinical records and may contain potentially sensitive clinical information.

## Supplementary material

SUPPLEMENTARY FIGURE S1 STROBE-style flow diagram of patient selection. A total of 218 medical records were screened during the study period. After exclusion of 138 records according to predefined eligibility criteria, 80 eyes from 80 patients were included in the final analysis. Exclusion categories were summarized according to the primary documented reason for exclusion.

## References

[1] Darabuş D-M Dărăbuş RG Munteanu M. The diagnosis and treatment of branch retinal vein occlusions: an update. Biomedicine. (2025) 13:105. doi: 10.3390/biomedicines13010105, 39857689 PMC11763247

[2] Chen K-Y Chan H-C Chan C-M. Effectiveness and safety of anti-vascular endothelial growth factor therapies for macular edema in retinal vein occlusion: a systematic review and network meta-analysis of randomized controlled trials. Surv Ophthalmol. (2025) 70:1067–89. doi: 10.1016/j.survophthal.2025.05.008, 40419166

[3] Fujihara-Mino A Mitamura Y Inomoto N Sano H Akaiwa K Semba K. Optical coherence tomography parameters predictive of visual outcome after anti-VEGF therapy for retinal vein occlusion. Clin Ophthalmol. (2016) 10:1305–13. doi: 10.2147/OPTH.S110793, 27486302 PMC4957686

[4] Modi YS Klufas MA Sridhar J Singh RP Yonekawa Y Pecen P. Current best clinical practices-Management of Retinal Vein Occlusion. J Vitreoretin Dis. (2020) 4:214–9. doi: 10.1177/2474126420906395, 37007445 PMC9982258

[5] Dinah C Dodds M Lotery A Salvatore S Fletcher E Lake AVR . Treatment patterns and long-term outcomes in anti-VEGF-treated macular oedema secondary to retinal vein occlusion: a retrospective observational study. Eye. (2026) 40:107–16. doi: 10.1038/s41433-025-04089-2, 41238745 PMC12764885

[6] Ciulla TA Hussain RM Taraborelli D Pollack JS Williams DF. Longer-term anti-VEGF therapy outcomes in Neovascular age-related macular degeneration, diabetic macular edema, and vein occlusion-related macular edema: clinical outcomes in 130 247 eyes. Ophthalmol Retina. (2022) 6:796–806. doi: 10.1016/j.oret.2022.03.021, 35381391

[7] Hatamnejad A Nanji K Grad J El-Sayes A Mihalache A Gemae M . Predicting treatment response in retinal vein occlusions using baseline optical coherence tomography biomarkers: a systematic review. Surv Ophthalmol. (2026) 71:100–18. doi: 10.1016/j.survophthal.2025.08.016, 40914443

[8] Tsang KK Hui VWK Pang CMK Tang Z Yang D Nguyen TX . SD-OCT-based biomarkers in predicting treatment outcomes of macular oedema secondary to retinal vein occlusion treated with anti-VEGF therapy. Acta Ophthalmol. (2026) 104:e152–64. doi: 10.1111/aos.17574, 40757884 PMC12888950

[9] Cunha Ferreira C Machado Soares R Fernandes J Teixeira S Saraiva E Ribeiro L . Predictive factors for functional and anatomical outcomes after anti-VEGF treatment for macular edema in patients with branch retinal vein occlusion. J Ophthalmic Vis Res. (2024) 19:324–33. doi: 10.18502/jovr.v19i3.13531, 39359524 PMC11443993

[10] Lai X-J Yang S-Y Lei C-Y Xiao R-H Zhang M-X. Correlations between inflammatory biomarkers in peripheral blood and branch retinal vein occlusion. Int J Ophthalmol. (2025) 18:1908–13. doi: 10.18240/ijo.2025.10.13, 40994607 PMC12454002

[11] Vural E Hazar L. Is the C reactive protein/albumin ratio a good tool for retinal vein occlusion? BMC Ophthalmol. (2025) 25:664. doi: 10.1186/s12886-025-04483-0, 41291569 PMC12648851

[12] Li X Yang L Long H Peng S. Comparative value of retinal structural features versus systemic inflammatory and renal indicators in predicting short-term anti-VEGF response in diabetic macular edema: focus on OCT biomarkers. Front Med. (2026) 13:1761324. doi: 10.3389/fmed.2026.1761324, 41709897 PMC12909192

[13] Ciulla T Pollack JS Williams DF. Visual acuity outcomes and anti-VEGF therapy intensity in macular oedema due to retinal vein occlusion: a real-world analysis of 15 613 patient eyes. Br J Ophthalmol. (2021) 105:1696–704. doi: 10.1136/bjophthalmol-2020-317337, 33055088 PMC8639936

[14] Liu JC Vatti T Seth K Valentim CCS Rachitskaya AV Singh RP. Outcomes in patients with retinal vein occlusion with good baseline visual acuity. Eye (Lond). (2023) 37:3203–8. doi: 10.1038/s41433-023-02488-x, 36949245 PMC10564869

[15] Kazantzis D Machairoudia G Kroupis C Theodossiadis G Theodossiadis P Chatziralli I. Complete blood count-derived inflammation indices and retinal vein occlusion: a case–control study. Ophthalmol Ther. (2022) 11:1241–9. doi: 10.1007/s40123-022-00511-0, 35503164 PMC9114275

[16] Bouchikh-El Jarroudi R Díaz-Aljaro PE Castellví-Manent J Romanic Bubalo N Fernandez-Torrón RD Sabala Llopart A . Predictive value of optical coherence tomography biomarkers on visual outcomes in patients with retinal vein occlusion. Ophthalmologica. (2026) 249:1–20. doi: 10.1159/000550350 41493899

[17] Ergin E Dascalu AM Stana D Tribus LC Arsene AL Nedea MI . Predictive role of complete blood count-derived inflammation indices and optical coherence tomography biomarkers for early response to intravitreal anti-VEGF in diabetic macular edema. Biomedicine. (2025) 13:1308. doi: 10.3390/biomedicines13061308, 40564027 PMC12189659

[18] Kushwaha P Singh A Shrivastava P. Inflammatory and hematologic biomarker profiles across central branch and hemi-retinal vein occlusion. Bioinformation. (2025) 21:2781–4. doi: 10.6026/973206300212781, 41393495 PMC12697520

