# Supplementary figures and images for: Baseline predictors of visual response and treatment burden after ranibizumab therapy in macular edema secondary to retinal vein occlusion
Source: Front Med (Lausanne). 2026 Jun 10;13:1856268. doi: 10.3389/fmed.2026.1856268 (PMC13290520; doi:10.3389/fmed.2026.1856268)

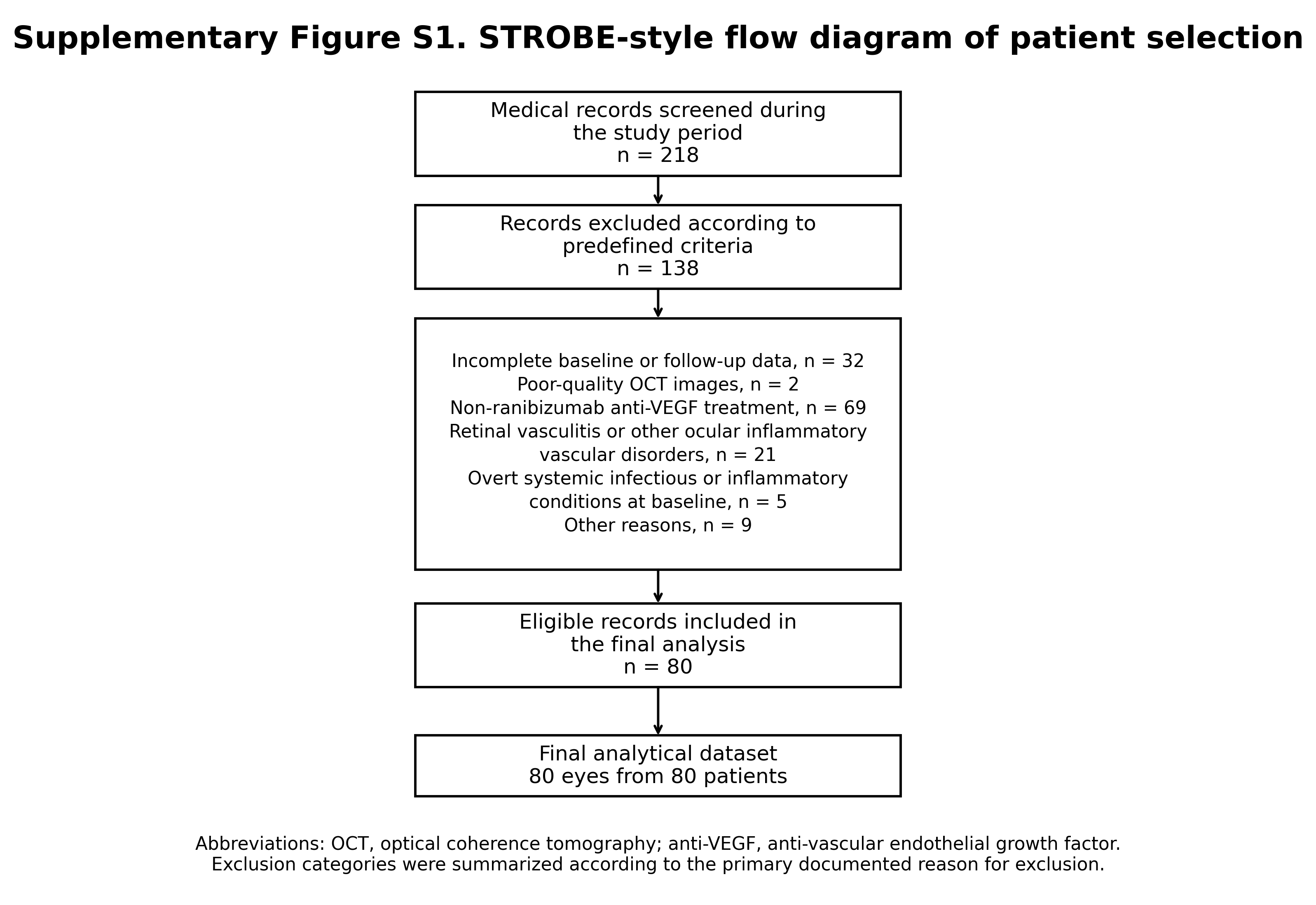

Supplement: SUPPLEMENTARY FIGURE S1 — STROBE-style flow diagram of patient selection. A total of 218 medical records were screened during the study period. After exclusion of 138 records according to predefined eligibility criteria, 80 eyes from 80 patients were included in the final analysis. Exclusion categories were summarized according to the primary documented reason for exclusion. [file Image_1.tif]
