# Supplementary material for: Baseline predictors of visual response and treatment burden after ranibizumab therapy in macular edema secondary to retinal vein occlusion
Source: Front Med (Lausanne). 2026 Jun 10;13:1856268. doi: 10.3389/fmed.2026.1856268 (PMC13290520; doi:10.3389/fmed.2026.1856268)
Supplement: Supplementary file 2 [file Supplementary_file_1.DOCX]

**Supplementary Table S1. Univariate logistic regression analysis for the composite functional endpoint**

| **Variable** | **OR** | **95% CI** | **P value** |
| --- | --- | --- | --- |
| Central macular thickness | 1.00 | 1.001–1.006 | 0.003 |
| RVO subtype: CRVO vs BRVO | 22.26 | 2.64–188.02 | 0.004 |
| Hypertension | 0.17 | 0.04–0.73 | 0.017 |
| PLR | 1.01 | 1.001–1.017 | 0.028 |
| Baseline ETDRS letters | 0.96 | 0.93–1.00 | 0.038 |
| NLR | 1.71 | 0.98–2.97 | 0.057 |
| SII | 1.00 | 1.000–1.004 | 0.059 |
| EZ/ELM disruption | 3.95 | 0.94–16.61 | 0.061 |
| Disease duration | 1.01 | 1.00–1.03 | 0.155 |
| MLR | 64.94 | 0.16–27102.34 | 0.175 |
| Male sex | 1.89 | 0.49–7.28 | 0.357 |
| DIRT | 1.78 | 0.46–6.87 | 0.402 |
| Diabetes mellitus | 0.44 | 0.05–3.81 | 0.459 |
| IRC | 1.65 | 0.39–6.92 | 0.493 |
| Age | 1.02 | 0.96–1.09 | 0.534 |
| SRF | 1.13 | 0.29–4.37 | 0.861 |
| RVO subtype: HRVO vs BRVO | Not estimable | Not estimable | - |

Footnote: Poor composite outcome was defined as failure to achieve either a gain of ≥10 ETDRS letters or a final decimal visual acuity of ≥0.3. BRVO was used as the reference category for RVO subtype. The HRVO estimate was not reported because of sparse events and unstable estimation.

Because only 10 eyes met the poor composite-outcome definition, these analyses were considered exploratory.

**Supplementary Table S2A. Spearman correlation analysis between baseline variables and the number of anti-VEGF injections within 6 months**

| **Variable** | **Spearman rho** | **P value** |
| --- | --- | --- |
| PLR | 0.240 | 0.032 |
| Baseline ETDRS letters | -0.229 | 0.041 |
| Central macular thickness | 0.215 | 0.055 |
| SII | 0.193 | 0.086 |
| Disease duration | 0.133 | 0.241 |
| NLR | 0.113 | 0.317 |
| MLR | -0.094 | 0.409 |
| Age | -0.018 | 0.877 |

Footnote: Spearman correlation analysis was used to evaluate associations between baseline continuous variables and injection number. These analyses were exploratory.

**Supplementary Table S2B. Comparisons of the number of anti-VEGF injections within 6 months across baseline categorical variables**

| **Variable** | **Category** | **Mean ± SD** | **Median** | **P value** |
| --- | --- | --- | --- | --- |
| RVO subtype | BRVO | 3.54 ± 0.68 | 3.0 | <0.001 |
|  | CRVO | 4.68 ± 0.48 | 5.0 |  |
|  | HRVO | 3.25 ± 0.50 | 3.0 |  |
| Hypertension | No | 4.33 ± 0.78 | 5.0 | 0.002 |
|  | Yes | 3.72 ± 0.77 | 4.0 |  |
| Diabetes mellitus | No | 3.89 ± 0.81 | 4.0 | 0.475 |
|  | Yes | 4.07 ± 0.88 | 4.0 |  |
| DIRT | 0 | 3.83 ± 0.85 | 4.0 | 0.280 |
|  | 1 | 4.03 ± 0.79 | 4.0 |  |
| EZ/ELM disruption | 0 | 3.85 ± 0.83 | 4.0 | 0.332 |
|  | 1 | 4.03 ± 0.81 | 4.0 |  |
| IRC | 0 | 3.78 ± 0.75 | 4.0 | 0.220 |
|  | 1 | 4.02 ± 0.86 | 4.0 |  |
| SRF | 0 | 3.90 ± 0.84 | 4.0 | 0.716 |
|  | 1 | 3.97 ± 0.81 | 4.0 |  |
| Disease duration | >30 days | 4.07 ± 0.83 | 4.0 | 0.235 |
|  | ≤30 days | 3.84 ± 0.82 | 4.0 |  |

Footnote: Descriptive data are shown as mean ± SD and median, whereas group comparisons were performed using the Kruskal-Wallis test or Mann-Whitney U test, as appropriate. These analyses were exploratory.

**Supplementary Table S3. Firth penalized logistic regression analysis for poor functional response after excluding HRVO eyes**

| **Variable** | **OR** | **95% CI** | **P value** |
| --- | --- | --- | --- |
| CRVO vs BRVO | 5.42 | 1.08–27.30 | 0.040 |
| Baseline ETDRS (per 10 letters) | 1.70 | 1.15–2.52 | 0.008 |
| Hypertension | 0.17 | 0.04–0.66 | 0.010 |
| Male sex | 4.38 | 1.21–15.93 | 0.025 |
| NLR | 1.43 | 0.87–2.36 | 0.162 |

Footnote: Firth penalized logistic regression was performed as a sensitivity analysis because of sparse-event concerns; n = 76.

Abbreviations: OR, odds ratio; CI, confidence interval; ETDRS, Early Treatment Diabetic Retinopathy Study; NLR, neutrophil-to-lymphocyte ratio.

**Supplementary Table S4. Multivariable linear regression analysis for final ETDRS letter score**

| **Variable** | **Beta** | **95% CI** | **P value** |
| --- | --- | --- | --- |
| Baseline ETDRS letters | 0.49 | 0.37 to 0.62 | <0.001 |
| CRVO vs BRVO | -13.78 | -19.62 to -7.93 | <0.001 |
| Hypertension | 3.62 | -1.62 to 8.85 | 0.173 |
| Male sex | -2.08 | -6.75 to 2.59 | 0.377 |
| CST (per 50 μm) | -0.40 | -0.87 to 0.06 | 0.088 |
| NLR | -2.07 | -4.19 to 0.05 | 0.056 |

Footnote: HRVO eyes were excluded from regression modeling; n = 76. Model fit: R² = 0.744; adjusted R² = 0.722.

Abbreviations: ETDRS, Early Treatment Diabetic Retinopathy Study; CI, confidence interval; CST, central macular thickness; NLR, neutrophil-to-lymphocyte ratio.

**Supplementary Table S5. Poisson regression analysis for the number of ranibizumab injections within 6 months**

| **Variable** | **IRR** | **95% CI** | **P value** |
| --- | --- | --- | --- |
| CRVO vs BRVO | 1.28 | 0.96–1.71 | 0.092 |
| Baseline ETDRS (per 10 letters) | 1.00 | 0.94–1.06 | 0.918 |
| CST (per 50 μm) | 1.00 | 0.98–1.02 | 0.991 |
| PLR (per 50 units) | 1.00 | 0.92–1.09 | 0.938 |
| Hypertension | 0.94 | 0.73–1.21 | 0.626 |

Footnote: HRVO eyes were excluded from regression modeling; n = 76. Injection count showed no evidence of overdispersion (mean = 3.96, variance = 0.68, variance-to-mean ratio = 0.17).

Abbreviations: IRR, incidence rate ratio; CI, confidence interval; ETDRS, Early Treatment Diabetic Retinopathy Study; CST, central macular thickness; PLR, platelet-to-lymphocyte ratio.

**Supplementary Table S6. Intergrader agreement for qualitative OCT biomarkers**

| **Biomarker** | **Agreements** | **Disagreements** | **% agreement** | **Cohen’s κ** | **Consensus positive n (%)** |
| --- | --- | --- | --- | --- | --- |
| DIRT | 76 | 4 | 95.0 | 0.899 | 38 (47.5%) |
| EZ/ELM disruption | 78 | 2 | 97.5 | 0.949 | 33 (41.2%) |
| IRC | 79 | 1 | 98.8 | 0.974 | 48 (60.0%) |
| SRF | 79 | 1 | 98.8 | 0.973 | 30 (37.5%) |

Footnote: Cohen’s κ was calculated between two masked retinal specialists; final consensus results were used in the main analyses; n = 80.

Abbreviations: OCT, optical coherence tomography; DIRT, disorganization of the inner retinal layers; EZ/ELM, ellipsoid zone/external limiting membrane; IRC, intraretinal cysts; SRF, subretinal fluid.
